# Supplementary material for: Effects of Temperature, Metal Ions and Biosurfactants on Interaction Mechanism between Caffeic Acid Phenethyl Ester and Hemoglobin
Source: Molecules. 2023 Apr 13;28(8):3440. doi: 10.3390/molecules28083440 (PMC10144779; doi:10.3390/molecules28083440)
Supplement: Supplementary file 1 [file molecules-28-03440-s001.zip › molecules-2266243-supplementary.pdf]

Supporting Information

For

Effects of Temperature, Metal Ions and Biosurfactants on Interaction Mechanism between Caffeic Acid Phenethyl Ester and Hemoglobin

Yutong Li[1], Zhen Zhao[2], Xiao Nai[1], Mingyuan Li[1], Jing Kong[1], Yanrong Chen[1], Min Liu[1], Qian Zhang[1], Jie Liu[1,*], Hui Yan[2,*]

[1]School of Chemistry and Chemical Engineering, Liaocheng University, Liaocheng 252059, China
[2]College of Pharmacy, Liaocheng University, Liaocheng 252059, China

*Corresponding authors. Tel.: +86 635 8230614, *E-mail address:* liujielcu@126.com (J. Liu) or yanhui@lcu.edu.cn (H. Yan)

Table S1. For the Hb and CAPE/NaC/NaDC system, the amino acid residues lining the binding site in Hb cavity, the hydrogen bonds, and the lowest binding energy.

Protein PDB	Small molecule	Surrounding amino acid residues	Hydrogen bonds	The lowest binding energy
carbonmonoxy liganded bovine Hb 1G09 (pH 7.2)	CAPE	THR134, THR137, LYS104, LYS99, SER133, ASN108, TYR35, HIS103, LEU105, ASP126, VAL96, ALA130, PRO95, PHE98, LEU129, SER102, LEU106	ASN108 (1.90 Å and 2.11 Å)<br>HIS103 (2.71 Å)<br>SER133 (1.65 Å)	-32.3 kJ/mol
	NaC	LYS99, PHE98, THR137, SER133, THR134, TYF140, SER138, VAL1, LYS139, AGR141, ASN131, LYS127, ALA130, VAL34, PRO36, TRP37<br>PRO35, VAL135	THR137 (1.73 Å)<br>THR134 (2.16 Å)<br>TYR140 (1.66 Å)<br>SER138 (1.86 Å)<br>LYS99 (1.73 Å and 1.92 Å)	-40.9 kJ/mol
	NaDC	LYS99, PHE98, THR137, SER133, THR134, TYP140, SER138, VAL1, LYS139, APG141, ASN131, LYS127, ALA130, VAL34, PRO36, TRP37<br>PRO95	THR137 (1.87 Å)<br>THR134 (2.27 Å)<br>TYR140 (1.62 Å)<br>SER138 (1.87 Å)<br>LYS99 (1.73 Å and 1.96 Å)	-41.1 kJ/mol
bovine deoxy Hb 1HDA	CAPE	VAL112, VAL109, HIS122, ASN108, LEU105, TRY35, ASP126, ALA130, LYS99, THR134, THR137, PRO95, SER133, VAL96, SER102, LEU129, LEU100, HIS103, LEU106	HIS103 (2.47 Å)<br>TYR35 (2.13 Å)<br>SER102 (1.82Å)<br>SER133 (1.79 Å)	-36.4 kJ/mol
	NaC	LYS99, ALA130, SER133, THR134, PRO95, THR137, SER138, TRY140, LYS139, TRP37, ARG141, LYS127, ALA130, VAL1	THR134 (2.16 Å)<br>THR137 (2.10 Å)<br>TYR140 (2.37 Å)<br>SER138 (2.72 Å)<br>ARG141 (1.67 Å)<br>LYS99 (1.61 Å)	-41.1 kJ/mol
	NaDC	LYS99, ALA130, SER133, THR134, PRO95, THR137, SER138, TRY140, LYS139, TRP37, ARG141, LYS127, ALA130, VAL1	THR134 (2.51 Å)<br>THR137 (2.21 Å)<br>TYR140 (1.91 Å)<br>SER138 (2.92 Å)<br>ARG141 (1.74 Å)<br>LYS99 (1.79 Å and 1.74 Å)	-41.9 kJ/mol
CO form of bovine Hb 6II1	CAPE	TRY145, ALA135, LYS132, VAL133, LEU3, LEU78, LEU81, GLY136, ASP79, ASP80, GLY83, LYS82, ALA140, ASN139, HIS146	LEU78 (2.11 Å)<br>ASP79 (2.85 Å)<br>LEU81 (2.12 Å)<br>LYS82 (1.80 Å)<br>HIS146 (1.84 Å)	-35.2 kJ/mol
	NaC	LYS104, VAL96, PRO100, ASP99, TRY145, THR38, LEU100, PHE36, ALA135, ALA138<br>PHE103, LYS99, THR39, PRO37, LYS132, SER35, GLN131, HIS103, VAL134, ASN108, GLY107, LEU105	SER35 (1.99 Å)<br>THR38 (2.00 Å)<br>ASN108 (2.10 Å)	-41.0 kJ/mol
	NaDC	LYS104, VAL96, PRO100, ASP99, TRY145, THR38, LEU100, PHE36, ALA135, ALA138<br>HIS146, ASN139, ALA142, ASN97, LYS104	ASP99 (1.84 Å)<br>LYS104 (1.75 Å and 1.75 Å)	-41.4 kJ/mol
carbonmonoxy liganded bovine Hb 1G08 (pH 5.0)	CAPE	GLN39, TRP37, PRO36, HIS89, LAA88,TYR140, THR137, LYS139, SER138, LYS127, ALA123, AGR141, SER124, ALA120, VAL34, PRO119, ALA51, THR50, VAL33, SER49, LEU48	LYS127 (2.189 Å)<br>SER108 (1.985 Å)<br>TYR140 (2.36 Å)	-36.0 kJ/mol

	NaC	VAL1, LEU2, ASN131, LYS127, ASP126, ALA123, VAL34, VAL33, PRO36, TYR140, SER138, LYS139<br>THR134, ALA130, TYR35, TRP37, TRY140, THR134	THR137 (2.16 Å)<br>SER138 (2.97 Å)<br>VAL34 (2.09 Å)<br>VAL1 (1.87 Å)<br>LYS127 (1.92 Å)<br>ASN131 (2.03 Å)	-40.2 kJ/mol
	NaDC	VAL1, LEU2, ASN131, LYS127, ASP126, ALA123, VAL34, VAL33, PRO36, TYR140, SER138, LYS139<br>AGR141, SER124, ASP6, SER3, PHE128	SER138 (2.46 Å)<br>ALA123 (2.13 Å)<br>VAL1 (2.20 Å)<br>LYS127 (1.72 Å)	-41.3 kJ/mol
carbonmonoxy liganded bovine Hb 1G0A (pH 8.5)	CAPE	VAL1, SER3, LYS127, ASP6, ALA5, GLY8, SER124, ASN9, VAL121, ALA120, PRO119, ALA123, VAL33, VAL34, PRO36, TRP37, LYS139, TYR140	ASP6 (1.81 Å)<br>ASN9 (2.68 Å)<br>ALA120 (2.25 Å)<br>SER124 (1.95 Å)<br>LYS139 (1.95 Å)<br>LYS127 (1.90 Å)	-38.8 kJ/mol
	NaC	VAL1, THR134, SER138, PRO77, LEU2, LYS7, ASP74, LEU73, ASP75, LEU76, VAL135, ASN131<br>SER3, ALA4, TYR140	LEU2 2.00 Å)<br>LYS7 (1.89 Å)<br>LEU73 (2.37 Å)<br>ASN131 (2.12 Å)<br>VAL1 (1.90 Å)	-41.7 kJ/mol
	NaDC	VAL1, THR134, SER138, PRO77, LEU2, LYS7, ASP74, LEU73, ASP75, LEU76, VAL135, ASN131	LYS7 (1.95 Å)<br>ASP74 (1.93 Å)<br>ASN131 (2.09 Å)	-42.3 kJ/mo

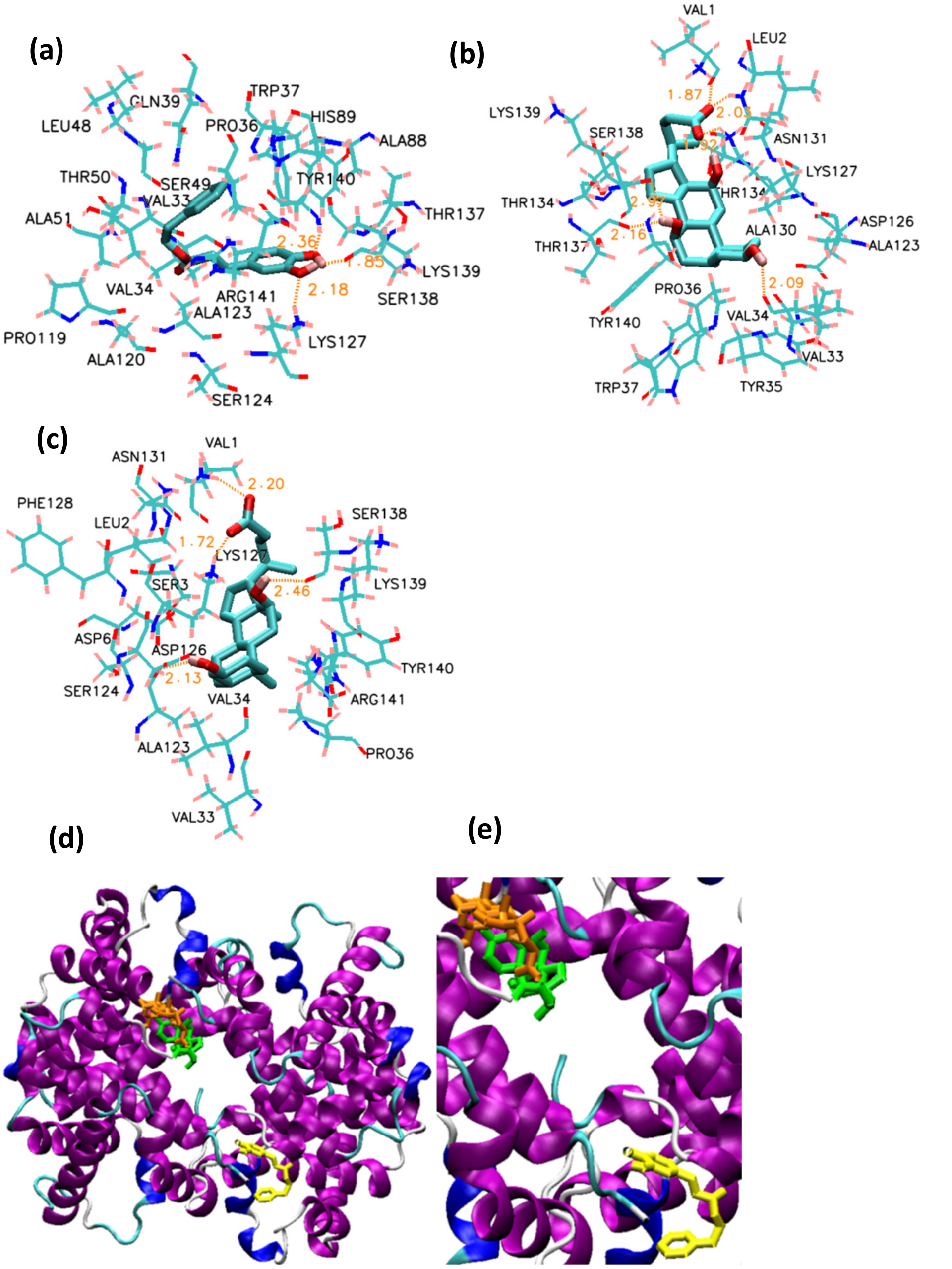

Figure S1. (a) CAPE and its surrounding amino acid residues for CAPE-Hb system; (b) NaC and its surrounding amino acid residues for NaC-Hb system; (c) NaDC and its surrounding amino acid residues for NaDC-Hb system; (d) The whole simulation diagram of Hb docking with CAPE, NaC and NaDC; (e) Partial enlargement of the Figure S1d. The spiral is the carbonmonoxy liganded bovine Hb (**PDB: 1G08 at pH 5.0**), the yellow rod is CAPE, the green rod is NaC, and the orange rod is NaDC.

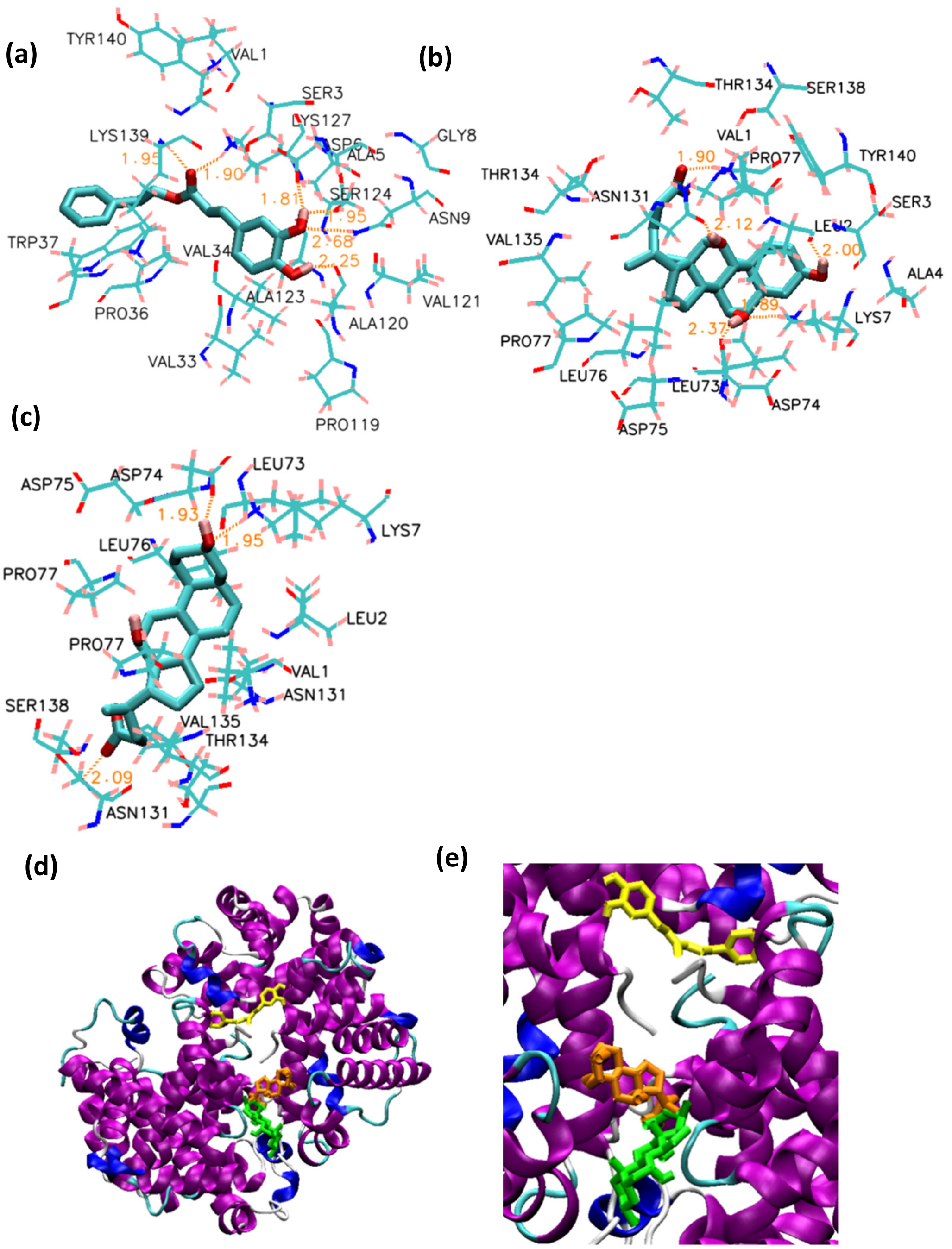

Figure S2. (a) CAPE and its surrounding amino acid residues for CAPE-Hb system; (b) NaC and its surrounding amino acid residues for NaC-Hb system; (c) NaDC and its surrounding amino acid residues for NaDC-Hb system; (d) The whole simulation diagram of Hb docking with CAPE, NaC and NaDC; (e) Partial enlargement of the Figure S2d. The spiral is the carbonmonoxy liganded bovine Hb (**PDB: 1G0A at pH 8.5**), the yellow rod is CAPE, the green rod is NaC, and the orange rod is NaDC.

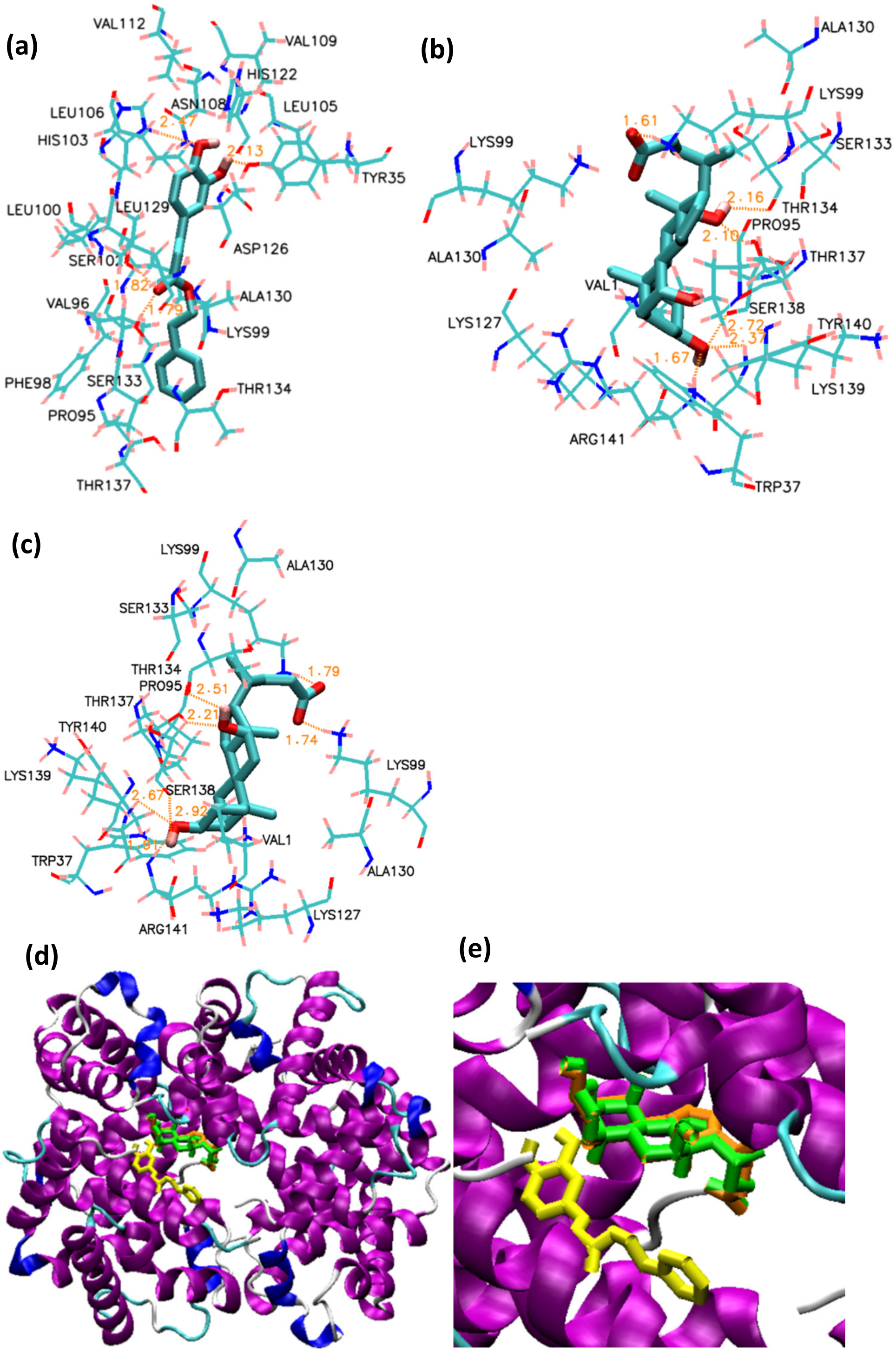

Figure S3. (a) CAPE and its surrounding amino acid residues for CAPE-Hb system; (b) NaC and its surrounding amino acid residues for NaC-Hb system; (c) NaDC and its surrounding amino acid residues for NaDC-Hb system; (d) The whole simulation diagram of Hb docking with CAPE, NaC and NaDC; (e) Partial enlargement of the Figure S3d. The spiral is the bovine deoxy Hb (**PDB ID: 1HDA**), the yellow rod is CAPE, the green rod is NaC, and the orange rod is NaDC.

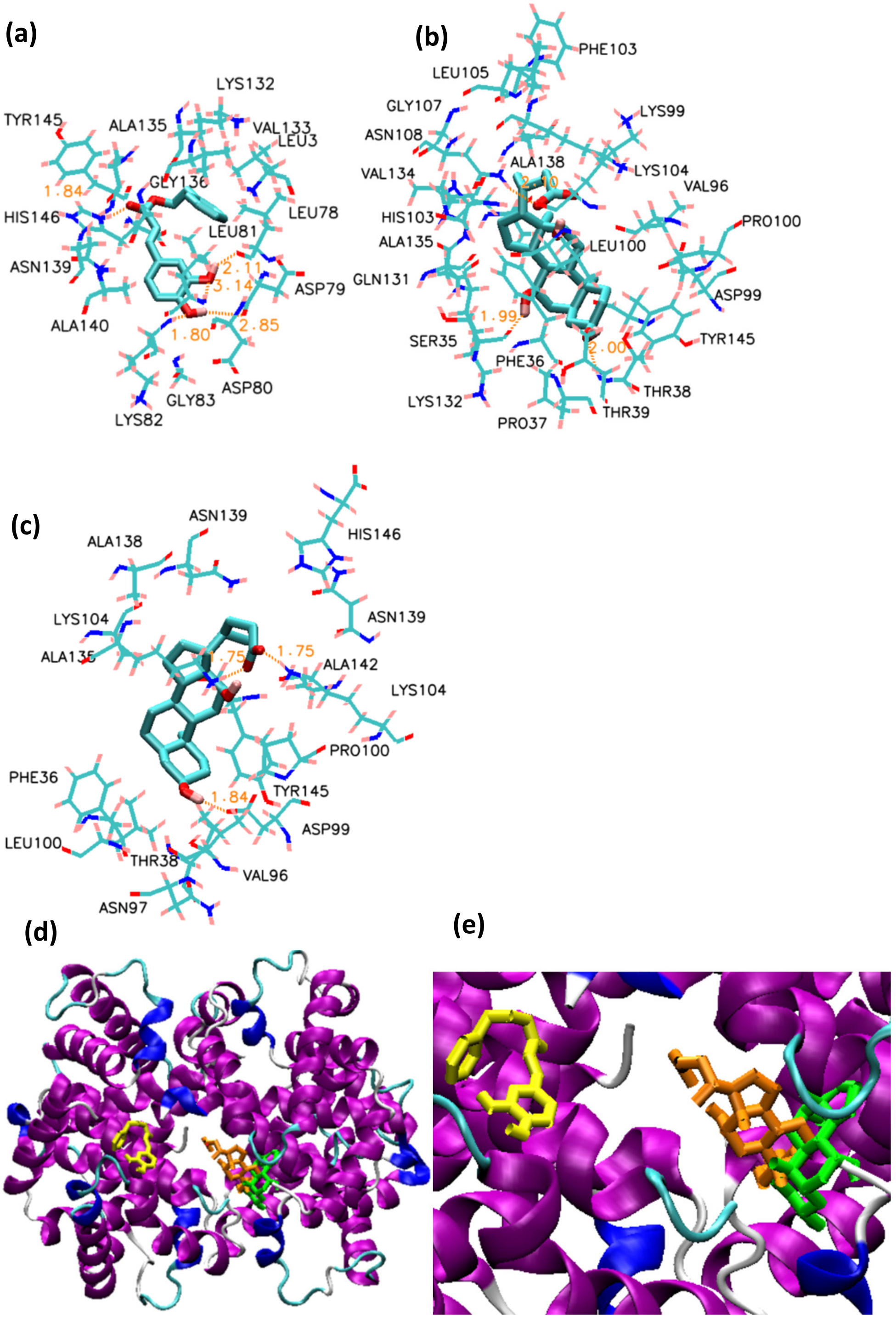

Figure S4. (a) CAPE and its surrounding amino acid residues for CAPE-Hb system; (b) NaC and its surrounding amino acid residues for NaC-Hb system; (c) NaDC and its surrounding amino acid residues for NaDC-Hb system; (d) The whole simulation diagram of Hb docking with CAPE, NaC and NaDC; (e) Partial enlargement of the Figure S4d. The spiral is the CO form of bovine Hb (**PDB ID: 6II1**), the yellow rod is CAPE, the green rod is NaC, and the orange rod is NaDC.

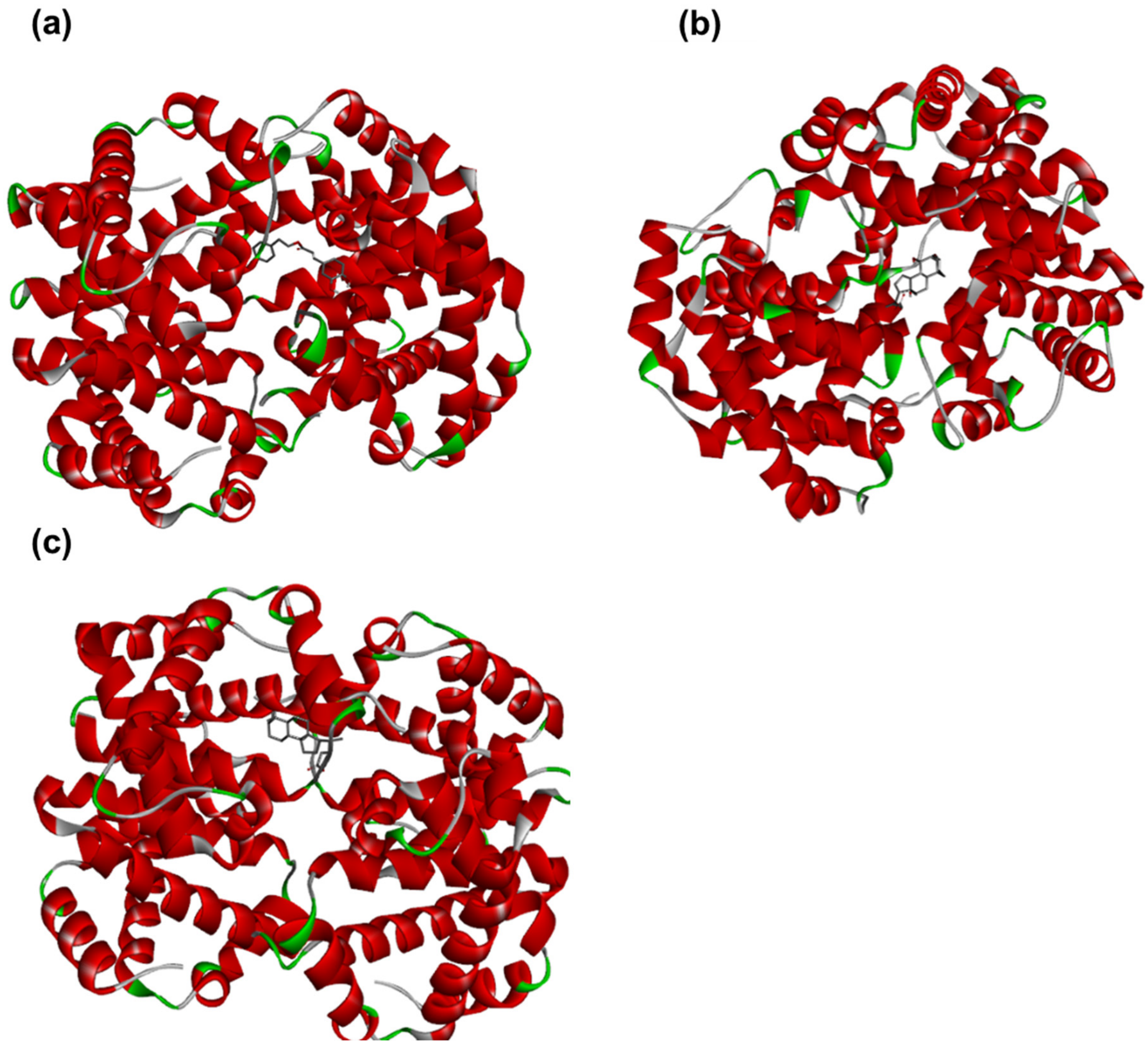

Figure S5. (a) Simulation diagram of molecular docking between Hb and CAPE; (b) Simulation diagram of molecular docking between Hb and NaC; (c) Simulation diagram of molecular docking between Hb and NaDC.
